# *In Vitro* Susceptibility to Closthioamide among Clinical and Reference Strains of Neisseria gonorrhoeae

**DOI:** 10.1128/AAC.00929-17

**Published:** 2017-09-22

**Authors:** Victoria F. Miari, Priya Solanki, Yonek Hleba, Richard A. Stabler, John T. Heap

**Affiliations:** a Faculty of Infectious & Tropical Diseases, London School of Hygiene & Tropical Medicine, London, United Kingdom; b Centre for Synthetic Biology and Innovation, Department of Life Sciences, Imperial College London, London, United Kingdom

**Keywords:** Neisseria gonorrhoeae, antimicrobial agents, closthioamide

## Abstract

Neisseria gonorrhoeae is one of the leading antimicrobial resistance threats worldwide. This study determined the MICs of closthioamide to be 0.008 to 0.5 mg/liter for clinical N. gonorrhoeae strains and related species. Cross-resistance with existing antimicrobial resistance was not detected, indicating that closthioamide could be used to treat drug-resistant N. gonorrhoeae.

## TEXT

Neisseria gonorrhoeae is one of the most important antimicrobial resistance (AMR) threats worldwide (1). Since the discovery of penicillin, N. gonorrhoeae has developed resistance to every therapeutic antimicrobial agent used (2).

Dual therapy for gonorrhea, with ceftriaxone and azithromycin, was introduced in 2011 (3) as a strategy to delay AMR. These antibiotics represent the last reliable classes of antibiotics recommended for the empirical treatment of N. gonorrhoeae infection (4), and worryingly, the MICs to both antibiotics are increasing annually (5). This is complicated further by reports of treatment failure due to extended-spectrum cephalosporin (ESC) resistance occurring worldwide.

In 2012, the World Health Organization (WHO) published an action plan to combat the spread and impact of N. gonorrhoeae (1). Given that there is no effective vaccine against gonorrhea and antimicrobial therapy is still one the most important means of gonorrhea control, the WHO advocates research into new antimicrobials (1).

Closthioamide (CTA), discovered in 2010, was isolated from the anaerobic bacterium Clostridium cellulolyticum (6). It represents a new class of natural polythioamide antibiotics and has been shown to have high *in vitro* activity against other AMR microorganisms (7). Its mode of action is thought to be by the inhibition of DNA gyrase (7). Cross-resistance to quinolone antibiotics has not been observed to date, suggesting a different mechanism of action (7). Given its high potency with multidrug-resistant (MDR) bacteria, closthioamide is a good candidate for testing against N. gonorrhoeae.

### Bacterial strains and antimicrobial susceptibility testing.

A total of 149 clinical and eight WHO reference N. gonorrhoeae isolates, as well as four commensal Neisseria strains, were examined in this study. These were provided by Barts Health NHS Trust, St George's University Hospitals NHS Foundation Trust, Royal Free NHS Foundation Trust, and Tunbridge Wells NHS Trust hospital laboratories. The MICs for CTA were determined by the agar dilution method as previously described (8). The CTA MIC range tested was 0.002 mg/liter to 1 mg/liter. Of the 149 clinical strains, the MICs to penicillin, ceftriaxone, azithromycin, ciprofloxacin, tetracycline, and spectinomycin had previously been determined for 131, and the MICs to cefixime were known for 127. The MICs of the WHO reference strains are shown in Table 1. Full MIC data for all of the antibiotics tested are provided in Table S1 in the supplemental material.

**TABLE 1:** MICs of WHO gonococcal reference strains^*a*^

WHO N. gonorrhoeae strain or breakpoint	MIC (mg/liter) for:							
	PEN	CFX	CRO	AZI	CIP	TET	SPE	CTA
F	0.032	<0.016	<0.002	0.125	0.004	0.25	32	0.063
G	0.5	<0.16	0.008	0.25	0.125	32	16	0.063
K	2	0.5	0.064	0.25	>32	2	16	0.5
L	2	0.25	0.125	0.5	>32	4	16	0.125
M	8	<0.016	0.012	0.25	2	1	16	0.125
N	8	<0.016	0.004	0.125	4	16	16	0.125
O	>32	0.016	0.032	0.25	0.008	1	>1,024	0.063
P	0.25	<0.016	0.004	2	0.004	0.5	16	0.125
MIC breakpoint	2	0.25	0.25	2	1	2	128	NA

a MICs determined by the World Health Organization (WHO) for penicillin (PEN), cefixime (CFX), ceftriaxone (CRO), azithromycin (AZI), ciprofloxacin (CIP), tetracycline (TET), spectinomycin (SPE). Closthioamide (CTA) MICs were determined by agar dilution in this study. NA, not applicable.

### CTA synthesis.

Closthioamide was synthesized according to the route described by Hertweck and coworkers (9, 10). The core of CTA was synthesized by two consecutive peptide couplings and deprotections onto a 1,3-diaminopropane core with an N-protected beta-alanine, followed by a third peptide coupling to install the aromatic benzoic acid end caps. Thionation with Lawesson's reagent and deprotection under highly acidic conditions yielded CTA in five longest linear steps.

All of the reagents involved in the synthesis of CTA were obtained from Sigma-Aldrich, with the exception of 1-ethyl-3-(3-dimethylaminopropyl)carbodiimide (EDCI), which was obtained from Manchester Organics. All of the reaction solvents used for the synthesis were anhydrous and of the highest grade from Sigma-Aldrich. All of the routine solvents for workup and purification were obtained from VWR. The closthioamide stock solution was prepared at 100 mg/liter in 100% ethanol.

### Statistical analysis.

MIC_50_ and MIC_90_ were calculated with MIC data from clinical gonococcal strains only. The correlation between MICs for CTA and those for other antibiotics was determined with a Spearman's rank correlation test using STATA 14.2. The correlation coefficient was calculated using MIC data from clinical and reference gonococcal strains.

### CTA susceptibility and cross-resistance to existing antimicrobials.

**FIG 1:**
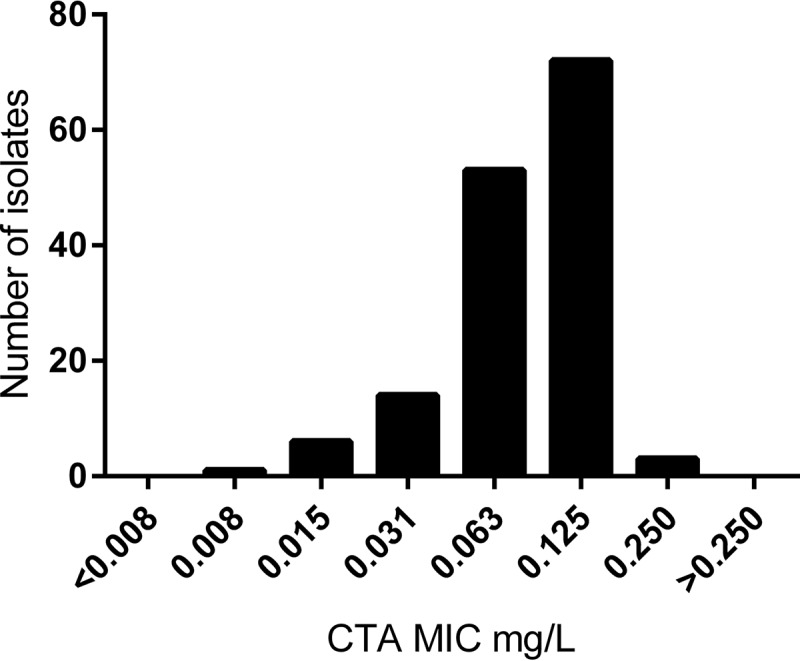
Susceptibility of gonococcal isolates to closthioamide (CTA). CTA was tested on 149 clinical gonococcal strains (range tested was 0.002 mg/liter to 1 mg/liter).

The CTA MICs for the 149 clinical strains ranged between 0.008 mg/liter and 0.25 mg/liter (Table S1). The numbers of isolates with MICs of 0.008 mg/liter, 0.015 mg/liter, 0.031 mg/liter, 0.063 mg/liter, 0.125 mg/liter, and 0.25 mg/liter were one (1%), six (4%), 14 (9%), 53 (36%), 72 (48%), and three (2%), respectively (Fig. 1). The MIC_50_ and MIC_90_ were 0.063 mg/liter and 0.125 mg/liter, respectively. The CTA MICs of N. lactamica and N. perflava were 0.063 mg/liter and 0.5 mg/liter, respectively, and both N. flavescens strains had MICs of >1 mg/liter.

**FIG 2:**
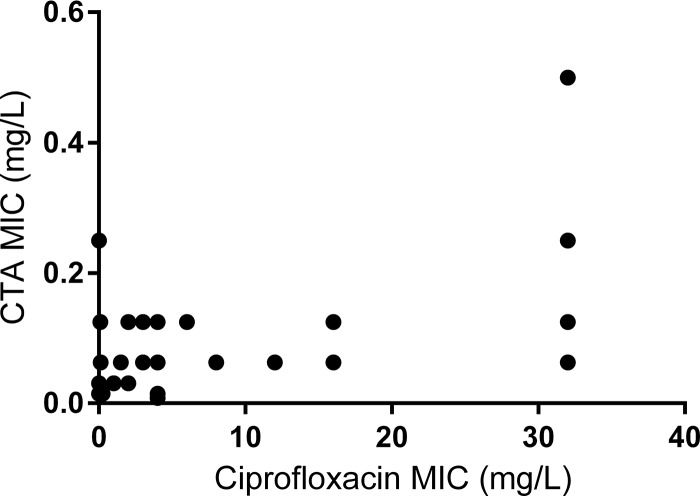
Correlation between CTA and ciprofloxacin MICs. MIC data for CTA and ciprofloxacin from 139 clinical strains were used to calculate a correlation coefficient (*R*^2^) of 0.07.

No significant correlation was identified between the MICs of the tested antibiotics. MICs for CTA and ciprofloxacin, a fluoroquinolone, had a correlation coefficient of 0.07 (Fig. 2). The numbers of isolates for each combination of CTA and ciprofloxacin MICs are shown in Table 2. MICs for azithromycin had the highest correlation with CTA MICs with an *R*^2^ 0.48.

**TABLE 2:** Number of clinical isolates with given combinations of CTA and ciprofloxacin MICs

Ciprofloxin MIC (mg/liter)	No. of isolates with CTA MIC (mg/liter) of:							Total no. of isolates
	0.008	0.015	0.031	0.063	0.125	0.25	0.5	
0.002	0	0	2	1	3	1	0	7
0.003	0	0	1	6	8	0	0	15
0.004	0	1	1	10	6	0	0	18
0.006	0	2	3	7	6	1	0	19
0.008	0	0	3	6	14	0	0	23
0.012	0	0	1	1	4	0	0	6
0.016	0	0	1	5	1	0	0	7
0.023	0	0	0	1	3	0	0	4
0.04	0	0	0	0	1	0	0	1
0.064	0	0	0	0	1	0	0	1
0.094	0	0	0	0	1	0	0	1
0.125	0	0	0	1	0	0	0	1
0.25	0	1	0	0	0	0	0	1
1	0	0	1	0	0	0	0	1
1.5	0	0	0	1	0	0	0	1
2	0	0	1	0	2	0	0	3
3	0	0	0	1	3	0	0	4
4	1	1	0	2	1	0	0	5
6	0	0	0	0	2	0	0	2
8	0	0	0	3	0	0	0	3
12	0	0	0	1	0	0	0	1
16	0	0	0	1	2	0	0	3
32	0	0	0	4	6	1	1	12
Total	1	5	14	51	64	3	1	139

The imminent threat of untreatable gonorrhea is a global problem that urgently requires the development of new antimicrobial agents. In this study, the novel antimicrobial CTA inhibited the growth of 146/149 (98%) clinical gonococcal strains at ≤0.125 mg/liter, suggesting the use of a low concentration for a therapeutic dose would reduce any potential toxicity. Importantly, isolates resistant to ciprofloxacin and the first-line therapeutic agents ceftriaxone and azithromycin are as susceptible to CTA as strains sensitive to these antibiotics, suggesting CTA could be effective clinically against MDR N. gonorrhoeae strains. Closthioamide activity against N. gonorrhoeae is comparable to its activity against other AMR organisms (7).

The CTA mode of action has been linked to DNA gyrase, which is also a target for fluoroquinolones, suggesting the potential for cross-resistance with antibiotics such as ciprofloxacin. However, the analysis of the clinical isolates demonstrated no correlation between MICs of the two antibiotics. This is also supported by a study by Chiriac et al., who found no cross-resistances between the two antimicrobials, although they did not examine N. gonorrhoeae (7). This suggests that the active site for CTA may be elsewhere in the quinolone resistance-determining region (QRDR).

Interestingly, the two N. perflava strains had the highest CTA MICs (>1 mg/liter), and the basis of this relative resistance requires further research to understand the specific resistance mechanisms.

In conclusion, CTA has high anti-gonococcal activity *in vitro*, even for multidrug-resistant isolates, but further studies to evaluate the clinical potential of this antimicrobial are urgently required in light of the public health threat that gonorrhea poses.

## Supplementary Material

Supplemental material
