# Supplementary material for: In Vitro Susceptibility to Closthioamide among Clinical and Reference Strains of Neisseria gonorrhoeae
Source: Antimicrob Agents Chemother. 2017 Sep 22;61(10):e00929-17. doi: 10.1128/AAC.00929-17 (PMC5610491; doi:10.1128/AAC.00929-17)
Supplement: Supplemental material [file supp_61_10_e00929-17__index.html]

Supplemental material 

# *In Vitro* Susceptibility to Closthioamide among Clinical and Reference Strains of Neisseria gonorrhoeae

## Supplemental material

- Supplemental file 1 -

  Supplemental Table S1

  XLSX, 19K
